# Editorial: Anticipatory Systems: Humans Meet Artificial Intelligence

**DOI:** 10.3389/fpsyg.2021.721879

**Published:** 2021-07-08

**Authors:** Mu-Yen Chen, Edwin Lughofer, Jose de Jesus Rubio, Yenchun Jim Wu

**Affiliations:** ^1^Department of Engineering Science, National Cheng Kung University, Tainan, Taiwan; ^2^Institute for Mathematical Methods in Medicine and Data Based Modeling, Johannes Kepler University of Linz, Linz, Austria; ^3^Sección de Estudios de Posgrado e Investigación, Escuela Superior de Ingeniería Mecánica y Eléctrica Azcapotzalco, Instituto Politécnico Nacional, Ciudad de México, Mexico; ^4^Graduate Institute of Global Business and Strategy, National Taiwan Normal University, Taipei City, Taiwan

**Keywords:** artificial intelligence, anticipatory systems, machine learning, deep learning, human-media interaction

Humans require encouragement the advances of computing paradigms. Some examples of this are ubiquitous computing presenting higher mobility, cloud computing yielding better functionality, and social computing providing better interactivity. Each of these examples introduces an implicit or explicit requirement from humans and attempts to realize these requirements through specific methods. However, humans may investigate more with the applications. A chatting robot is one example, as this robot is used to maintain relationships with our social contacts when we do not have sufficient space or time to do so. To this end, it continuously interacts with our contacts by computing our thinking patterns, behaviors, and other important information. Of course, there are many interesting studies in this topic, which invites the discussion of a new computing paradigm, “Anticipatory Computing.” This computing paradigm indicates a topic related to applications developed and able to anticipate specific user requirements. It is also utilized together with novel applications to perform an action in anticipation of a question of the user or to send a suggestion to the user. This is not only an example of artificial intelligence, it is one example of an innovation (i.e., prediction plus action). It can also be expressed as key to developing well-being within society, and a way to reach the ideal of “serve before you ask.” This phenomenon will be considered as an opportunity to raise challenging problems within the topic of computer science.

Taking into account the invaluable crowd intelligence residing in the social network and big data content, opportunities emerge to enable promising smart technology to find individual requirements, generate company business models, and suggest optimal life development. Hence, the nature of big data also provides important challenges from multiple perspectives to methods and technology that rely on social big data. These consider algorithm effectiveness, computation speed, energy efficiency, user privacy, server security, and system scalability. The main goal of this Research Topic is to collect 14 manuscripts reporting original contributions related to deep neural networks or machine learning methods for building anticipatory systems.

The paper titled “A Book Interaction Scheme to Enhance Children's Reading Experiences and Preferences” by Zhang et al. researches the interaction between a book and 5–6 years old children, considering reading choices, measuring reading time, and emotional answer to increase their reading knowledge, and developing books in relation to these interactions.

The paper titled “A priori Algorithm for the Data Mining of Global Cyberspace Security Issues for Human Participatory Based on Association Rules” by Li et al. is focused on the a priori approach in association rules; consequently, a total of 181 relevant rules are mined from 40 objective websites and 56,096 web pages are related with cyberspace security; in addition, this paper studies the support, trust, promotion, leverage, and reliability to reach a comprehensive data set.

The paper titled “Exploration of Social Benefits for Tourism Performing Arts Industrialization in Culture-Tourism Integration Based on Deep Learning and Artificial Intelligence Technology” by Zhang, studies the social advantage of the culture-tourism factory; hence, an intelligent model is built for the social advantage evaluation based on the descending gradient neural network and fuzzy model, considering the Yiyang Town as an example.

In the paper titled “Early Warning Method for Public Health Emergency Under Artificial Neural Network in the Context of Deep Learning” by Zheng and Hu, the objective is to decrease the substantial losses produced by public health emergencies in people; hence, the tuberculosis data from June 2017 to 2019 in a city are obtained, and the forecast model is built using the Convolutional Neural Network and Artificial Neural Network.

The paper titled “Measuring and Improving User Experience Through Artificial Intelligence-Aided Design” by Yang et al. suggests to collect user behavior data from logs of a mobile technology; in order to provide the privacy of users, the information is utilized to process the browsing and performing of mobile technology, the target of the proposed technique is to design a deep neural network model to simulate the user's experience in the processing of a mobile technology.

In the paper titled “The Talent Training Mode of International Service Design Using a Human-Computer Interaction Intelligent Service Robot From the Perspective of Cognitive Psychology” by Yang, the human-computer interaction (HCI) application and artificial intelligence are utilized to build the international service talent learning mode of the HCI intelligent service robot, this mode can be utilized to solve the existing teaching issues by utilizing new means to assure the quality of teaching.

In the paper titled “Can Students' Computer Programming Learning Motivation and Effectiveness Be Enhanced by Learning Python Language? A Multi-Group Analysis” by Ling et al., the main goal is to research about the Python algorithm performance on students' computing training; the junior students of two types in a college are the investigation participants, the training results in the Java algorithm and Python algorithm are compared to show the differences.

In the paper titled “A Meaning-Aware Cultural Tourism Intelligent Navigation System Based on Anticipatory Calculation” by Meng and Liu utilizes the theory of desired behavior to obtain the relation between tourists' attitude, experience behavior, and shows the information usage by taking the purple clay culture presentation as an example.

In the paper titled “Developing an Instrument for Assessing Self-Efficacy in Data Mining and Analysis” by Wang et al., the purpose is to design a device for evaluating self-efficacy in data mining and analysis based on the skills and abilities about performing data mining and study, and expert suggestions, the introduced device is important in evaluating the individual's abilities in performing data mining.

In the paper titled “Design and Implementation of Intelligent Sports Training System for College Students' Mental Health Education” by Wang and Park, an intelligent sports management model based on deep training method is developed by utilizing human-computer interaction and information application in artificial intelligence to determine the issues of poor physical fitness of university students and the low accuracy of university sport venues' performance.

In the paper titled “The Use of Deep Learning and VR Technology in Film and Television Production From the Perspective of Audience Psychology” by Tong et al., the design of artificial intelligence (AI) application, the deep-training (DT)-based Virtual Reality (VR) application, and DT application are used in human-computer interaction (HCI), and their results on TV works production, modern film, and audience thinking are analyzed.

The paper titled “Exploring the Influential Factors on Readers' Continuance Intentions of E-Book APPs: Personalization, Usefulness, Playfulness, and Satisfaction” by Liu et al. is focused on the influential factors on university students' continuance intention of e-book technology, this studied literature corresponds to the electronic book (e-book) technology and university students' continuous intention, and presents the reasons that encourage university students' continuous intention of using e-book technology.

In the paper titled “Exploring Computational Thinking Skills Training Through Augmented Reality and AIoT Learning” by Lin et al., a new AIoT training with Augmented Reality (AR) application is introduced and the results of CT skills are analyzed, the graduate utilizes AR technology to understand AIoT technology in application, considers the set of different AR sensors in recent scenarios, further generalization, and developed programs.

The paper titled “Attention-Based Deep Entropy Active Learning Using Lexical Algorithm for Mental Health Treatment” by Ahmed et al. is focused on the technology of individualized mental health interventions by utilizing the Natural Language Processing (NLP) and attention-based in-depth entropy active training; for this goal, authors introduce a technique based on synonym expansion by semantic vectors.

Last but not least, we finish this editorial by thanking all authors for their proposals to this Research Topic and to all reviewers for their voluntary revisions in the peer-review process to maintain a high standard of our Research Topic.

## Author Contributions

M-YC and JR: writing—original draft. EL and YW: review and editing. All authors have read and agreed to the published version of the paper.

## Conflict of Interest

The authors declare that the research was conducted in the absence of any commercial or financial relationships that could be construed as a potential conflict of interest.

